# Preservation of Retinal Function Through Synaptic Stabilization in Alzheimer's Disease Model Mouse Retina by Lycium Barbarum Extracts

**DOI:** 10.3389/fnagi.2021.788798

**Published:** 2022-01-13

**Authors:** Jinfeng Liu, Larry Baum, Shasha Yu, Youhong Lin, Guoying Xiong, Raymond Chuen-Chung Chang, Kwok Fai So, Kin Chiu

**Affiliations:** ^1^Department of Ophthalmology, LKS Faculty of Medicine, The University of Hong Kong, Pokfulam, Hong Kong SAR, China; ^2^Department of Psychiatry, LKS Faculty of Medicine, The University of Hong Kong, Pokfulam, Hong Kong SAR, China; ^3^State Key Laboratory of Brain and Cognitive Sciences, The University of Hong Kong, Pokfulam, Hong Kong SAR, China; ^4^Tianjin Key Lab of Ophthalmology and Visual Science, Clinical College of Ophthalmology, Tianjin Eye Hospital, Nankai University Eye Hospital, Tianjin Eye Institute, Tianjin Medical University, Tianjin, China; ^5^Laboratory of Neurodegenerative Diseases, School of Biomedical Sciences, The University of Hong Kong, Pokfulam, Hong Kong SAR, China; ^6^Department of Psychology, The University of Hong Kong, Pokfulam, Hong Kong SAR, China; ^7^Guangdong-Hongkong-Macau Institute of CNS Regeneration, Jinan University, Guangzhou, China

**Keywords:** Alzheimer's disease, retina, synapse, Lycium barbarum, 8-OHG, calpain-2, calpain-5

## Abstract

In Alzheimer's disease (AD), amyloid β deposition-induced hippocampal synaptic dysfunction generally begins prior to neuronal degeneration and memory impairment. *Lycium barbarum* extracts (LBE) have been demonstrated to be neuroprotective in various animal models of neurodegeneration. In this study, we aimed to investigate the effects of LBE on the synapse loss in AD through the avenue of the retina in a triple transgenic mouse model of AD (3xTg-AD). We fed 3xTg-AD mice with low (200 mg/kg) or high (2 g/kg) dose hydrophilic LBE daily for 2 months from the starting age of 4- or 6-month-old. For those started at 6 month age, at 1 month (though not 2 months) after starting treatment, mice given high dose LBE showed a significant increase of a wave and b wave in scotopic ERG. After 2 months of treatment with high dose LBE, calpain-2, calpain-5, and the oxidative RNA marker 8-OHG were downregulated, and presynaptic densities in the inner plexiform layer but not the outer plexiform layer of the retina were significantly increased, suggesting the presynaptic structure of retina was preserved. Our results indicate that LBE feeding may preserve synapse stability in the retina of 3xTg-AD mice, probably by decreasing both oxidative stress and intracellular calcium influx. Thus, LBE might have potential as a neuroprotectant for AD through synapse preservation.

## Introduction

The number of people with dementia is about 55 million worldwide in 2021, and is expected to increase to about 140 million by 2050 (World Health Organization., 2021). Alzheimer's disease (AD) is the most common type of dementia (60–70% of cases). It is an evolving humanitarian challenge, with growing numbers due to the aging of the population. In the United States alone, the estimated total healthcare cost for the treatment of AD in 2020 was estimated at $305 billion, and is estimated to increase to more than $1 trillion by 2050 (Wong, 2020). Early detection and diagnosis of AD, leading to early initiation of current AD therapies, is associated with improved quality of life and economic outcomes. This is despite the modest effectiveness of current AD therapies, therefore finding an improved and affordable treatment would be very important for society.

Transgenic mouse models harboring mutated human genes associated with familial forms of AD are advanced preclinical tools in the study of mechanisms underlying AD. The triple transgenic mouse model of AD (3xTg-AD) incorporates a Swedish amyloid precursor protein (APP) mutation, a human mutant presenilin 1 (PSEN1) gene PS1 (M146V) knock-in, and a tau (P301L) transgene (Oddo et al., 2003). These mice develop intracellular amyloid beta (Aβ), Aβ plaques and neurofibrillary tangle (NFT) like pathology in a progressive and age-dependent manner similar to human AD (Oddo et al., 2003; Edwards et al., 2014). AD disproportionately affects women in both disease prevalence and rate of symptom progression (reviewed by Fisher et al., 2018). 3xTg-AD mice also display faster progression in females than in males. Starting at 6 months of age, female 3xTg-AD mice exhibit greater cognitive deficits than males. This disparity is still evident at 9 months of age, when female mice have significantly higher stress responses (Clinton et al., 2007). Thus, it is crucial to specify sex and age to examine early-stage AD mechanisms and/or novel therapeutic interventions.

In the early stage of AD, memory impairment and loss of hippocampal synapses show up prior to frank neuronal degeneration (Selkoe, 2002; Chen et al., 2019). In early stages of familial AD, neuronal RNA oxidation occurs (Nunomura et al., 2004, 2009; Nunomura and Perry, 2020). Increased oxidative stress (Xu et al., 2021), uncontrolled hyperactivation of a family of cysteine proteases called calpains (Mahaman et al., 2019), and synaptic dysfunction (Jackson et al., 2019) may contribute to the disease process. The abnormal activation of calpain could induce synaptic dysfunction (Chen et al., 2019). Calpain inhibition not only prevented Aβ (1-42) induced Ca^++^ influx and neuronal death in primary cortical neuron culture (Lee et al., 2000), but also decreased the AD-like pathology and cognitive decline in aged 3xTg-AD mice (Medeiros et al., 2012). Mounting evidence indicates that therapeutic approaches aiming to protect against these in the early stage of AD might stop or reverse disease progression (Jackson et al., 2019).

Emerging evidence suggests that visual performance is impaired early in AD (Van Wijngaarden et al., 2017; reviewed by Shah et al., 2017). Visual impairments reported in AD patients include nerve fiber layer thinning, degeneration of retinal ganglion cells (RGC), and changes to vascular parameters (Hart et al., 2016). Moreover, Aβ deposits were observed in multiple layers of the retina, and were significantly associated with brain Aβ burden. The retina (and its related vasculature and optic nerve) shares the same embryological origin as well as some anatomical and physiological properties as the brain (Patton et al., 2005). In transgenic AD animals, retinal abnormalities demonstrated by deterioration in visual function or reactive gliosis were reported (Chidlow et al., 2017; Chiquita et al., 2019a; Zhang et al., 2021). Any neuroprotective agents benefitting the visual system might be useful for AD treatment.

Lycium barbarum (LB) is named Gouqizi in Chinese, and wolfberry in English. It has been used medicinally for more than 2500 years for its abilities to delay aging and improve visual acuity. LB has been regularly consumed by a vast number of people, with little to no side effects, making it a good candidate for an anti-aging agent (Gao et al., 2017). The neuroprotective effect of LB was first demonstrated in animal models of retinal degenerative diseases such as glaucoma, age-related macular degeneration (ARMD) (Chiu et al., 2009). It appears to protect the visual system through four primary processes: neuroprotection, blood-retinal barrier stabilization, antioxidation, and modulation of retinal immune function via the retinal microglial cells and Müller cells (reviewed by Manthey et al., 2017). LB can inhibit two key pro-apoptotic signaling pathways (JNK and PKR) in Aβ peptide neurotoxicity (Chang et al., 2002; Suen et al., 2003; Yu et al., 2005, 2006). The neuroprotective effects of LB were further demonstrated in the preservation of cognitive functions and decrease in Aβ deposition in APPswe/PS1-del9 Tg-AD mice (Zhang et al., 2013; Zhou et al., 2020).

Studies using extracts revealed the beneficial effects of LB. The major LB constituents demonstrating anti-aging properties include LB polysaccharides, carotenoids (zeaxanthin and β-carotene), betaine, flavonoids and vitamins (Gao et al., 2017). Our previous study using hydrophilic extract from LB (LBE) demonstrated the enhancement of retinal light response in the retina of 5xFAD mice (Zhang et al., 2021). Further study in cell culture using the IMG microglial cell line showed that LBE promoted activation of microglia with anti-inflammatory character (M2 polarization) and reduced oligomeric Aβ induced inflammatory reactions in microglia (Sun et al., 2022). LBE also had antioxidant effects under H_2_O_2_ stimulation of primary mixed glial cells (Zheng et al., 2021). Therefore, if LBE is applied early in AD, disease progression might be significantly delayed.

In the current study, we aimed to explore the effects of LBE feeding on the retinal changes in young female 3xTg AD mice, and to unveil the underlying mechanisms. The evaluation was focused on retinal function changes and RNA oxidation, calpain activation and synaptic proteins in the retina. Because much of retinal function and pathology can be observed non-invasively in living animals and humans, the ability to evaluate treatment effects in the retina would be an invaluable and low cost translational research platform to fulfill an unmet need of healthy aging.

## Materials and Methods

### Animals

3xTg-AD mice [B6;129-Psen1tm1Mpm Tg (APPSwe, tauP301L)1Lfa/J] (Oddo et al., 2003) were purchased from the Jackson Laboratory (stock No. 004807, Bar Harbor, ME, USA). Since the mice are homozygous for mutations in the PS1, APP, and tau genes, we maintained the colony by breeding homozygous 3xTg-AD mice to each other. Non-transgenic C57BL/6J mice were obtained from the Laboratory Animal Unit of the University of Hong Kong and used as wild-type (WT) controls. To reduce sexual dimorphism, especially in the early stage of AD (from 4 to 8 months), only female mice were used in the study. All animals were maintained in a temperature-controlled room with a 12-h light/dark cycle throughout the observation period. All animal procedures were performed according to the ARRIVE guidelines and were approved by the Committee on the Use of Live Animals in Teaching and Research of the University of Hong Kong. All efforts were taken to minimize the number of animals used and their suffering.

### LBE Preparation, Animal Feeding and Grouping

LBE was provided by Eu Yan Sang (HK) Ltd. LB from NingXia Huizu Autonomous Region, the People's Republic of China, was used. The extraction procedure was the same as in our previous report (Sun et al., 2022). Briefly, 2.5 kg of LB was washed and soaked in 40°C ultrapure water for 15 minutes. After 1 h boiling, the filtered drug residues were boiled again. The extracts from two boils were combined and concentrated to 1.25 kg. The final LBE was weighed and diluted in ultrapure water (w/v) to produce a stock solution (100 g/L).

3xTg-AD mice were orally fed with a low dose (200 mg/kg body weight) or high dose (2 g/kg) of LBE daily for 2 months with distilled water feeding as vehicle control, and age-matched C57BL/6J mice with the same treatments were regarded as WT controls. For each age stage (4 and 6 months old), five groups were included: water treated WT mice, high dose LBE treated WT mice, water treated AD mice, low dose LBE treated AD mice, and high dose LBE treated AD mice. Each group consisted of six mice.

### Flash Electroretinography

Retinal function was evaluated by an electroretinography (ERG) system (Espion E2 Electrophysiology System, Diagnosys LLC, USA) according to the standard protocol of the International Society for Clinical Electrophysiology of Vision (ISCEV). Mice were anesthetized with intraperitoneal injection of a mixture of ketamine (0.1 mg/g) and xylazine (10 μg/g). The eyes were administered 1% Mydriacyl (Alcon, Fort Worth, USA) to dilate the pupils and 0.5% Alcaine (Alcon, Fort Worth, USA) to reduce cornea sensitivity. ERG signals under a scotopic flash intensity of 3.0 cd·s/m^2^ and photopic flash intensity of 22.8 cd·s/m^2^ were recorded. Amplitude and latency of ERG signals were filtered and analyzed by Axon pCLAMP 10 (Molecular Devices Corp., Sunnyvale, CA, USA).

### Histology and Morphometric Analysis of Retinal Sections

Mice were sacrificed by an overdose of anesthesia with an intraperitoneal injection of Dorminal (0.1–0.15 mg/g). The eyeballs were harvested, fixed, processed and paraffin-embedded for sectioning. Five micrometer thick retinal cross sections with intact optic nerves were deparaffinized and stained with hematoxylin and eosin. Images were captured with a light microscope (Eclipse80i, Nikon) under 40x magnification. Morphometric analysis was carried out as in our previous report (Chan et al., 2012). The thickness of the inner retinal layer (IRL) was measured from inner limiting membrane to the outermost point of the inner nuclear layer (INL), and the outer nuclear layer (ONL), as well as the number of RGC both in the central or peripheral retina, were calculated using Fiji software (NIH, MD, USA).

### Immunohistochemical Detection in Retinal Sections

Retinal sections were rehydrated and boiled in 95°C citric acid buffer (10 mM, pH 6.0) for 15 min for antigen retrieval. Sections were then incubated in 10% normal goat serum with 1% bovine serum albumin (Sigma-Aldrich, St. Louis, MO, USA) and 0.1% Triton X-100 (Sigma-Aldrich, St. Louis, MO, USA) in PBS (pH 7.2–7.4) for 1 h at room temperature to block nonspecific binding. Following blocking, sections were incubated with either of the following primary antibodies overnight at 4°C: anti-goat Brn3a (1:500, Santa Cruz, Dallas, USA); anti-mouse PKC-α (1:500, Millipore, Burlington, Massachusetts, USA); anti-rabbit GFAP (1:500, Abcam, Cambridge, UK); anti-rabbit Iba-1(1:500, WAKO, Chou-ku, Osaka, Japan); anti-rabbit synaptophysin (1:500, Abcam); anti-rabbit PSD-95 (1:500, Abcam); anti-rabbit 8-OHG (1:500, Abcam); anti-rabbit calpain-2 (1:200, Thermo Fisher Scientific, Waltham, Massachusetts, USA); or anti-rabbit calpain-5 (1:200, Thermo Fisher Scientific). After washing with PBS, the sections were incubated with Alexa-568 or 488 fluorescent-conjugated goat IgG secondary antibody (1:500; Thermo Fisher Scientific) for 1 h at room temperature. The slides were then washed in PBS and counterstained with 4',6-Diamidino-2-phenylindole (DAPI) (1:1000). Images were captured using a ZEISS LSM 800 Confocal microscope (Carl Zeiss Microscopy GmbH, Germany).

### Statistical Analysis

Statistical analysis was performed by *t*-test or one-way ANOVA followed by *post-hoc* analysis using SPSS software for Windows (version 19.0; SPSS, Inc., IL, USA). Data were reported as mean ± SD. A value of P < 0.05 was considered significant for statistical analysis.

## Results

### Age Dependent Retinal Functional Impairment and Accumulation of Intracellular Amyloid in the 3xTg-AD Mice

**Figure 1:**
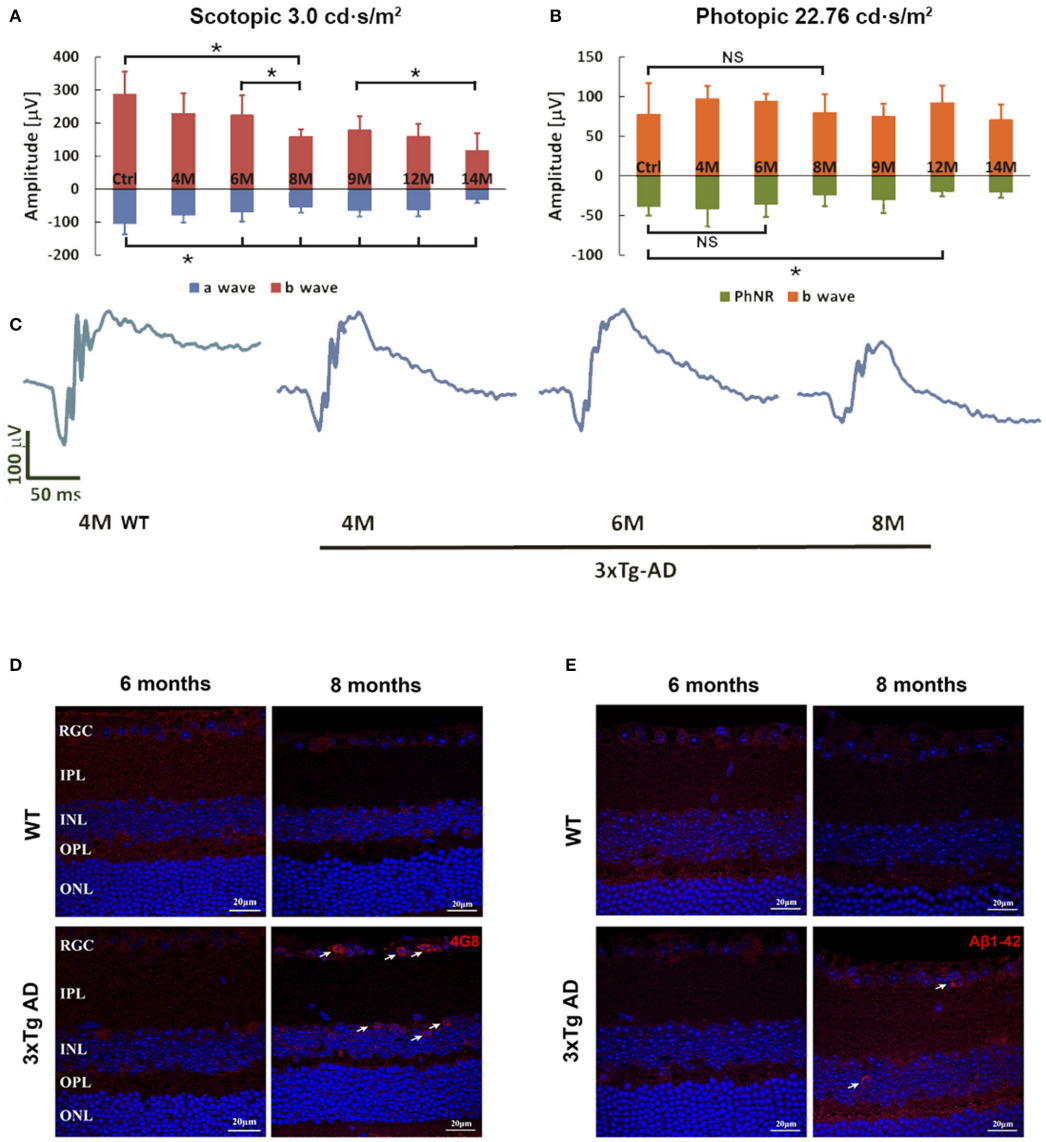
Flash-ERG and amyloid beta (Aβ) deposition in retinas of non-Tg control mice and 3xTg-AD mice at different ages. The scotopic retinal response **(A)** of 3xTg-AD mice started to decline at 6 months of age, followed by a rapid decrease from 6 to 8 months of age **(A,C)**. *p < 0.05. Further significant decline in b wave changes can be detected between 6–8 months and 9–14 months. Significant reduction in a wave started at 6 months when compared with non-Tg controls. PhNR detected in the photopic response revealed a significant reduction at 12 months when compared with non-Tg controls **(B)**. *p < 0.05; NS, no significant change. Typical ERG wave forms are shown in C. Intracellular Aβ deposition of 4G8 **(D)** and Aβ1-42 **(E)** were detected in the RGCL and INL of retinas of 3xTg-AD mice at 8 months only (arrows). No positive signal could be detected in the non-Tg controls or 6 month old 3xTg-AD retinas. Scale bar: 20 μm.

Retinal function reflected by ERG parameters demonstrated age dependent changes from 4 to 14 months (Figure 1). Compared with WT control, the scotopic retinal response in a wave started to decline significantly at age 6 months, followed by a rapid decline between 6 and 8 months (Figures 1A,C). Significant decline in b wave could be detected between 6 to 8 months and 9 to 14 months in 3xTg-AD mice. Under light adaptation, the changes in b wave were not significant when compared to WT control. There was significant PhNR reduction at 12 months of age (Figure 1B, ^*^p < 0.05). Deterioration of retinal function in ERG might be caused by decreased neuronal activity or connectivity or by loss of retinal neurons. At the age of 8 months, morphometric comparison of the thickness of IRL and ONL, and number of RGC in H&E-stained retinal sections (Supplementary Figure S1), did not show any significant changes between WT and 3xTg-AD mice. Further immunohistochemical detection and counting of Brn-3a positive RGCs, PKC-α labeled retinal bipolar cells and GFAP labeled astrocytes and active Müller cells also confirmed that there was no significant change in the cell densities at the age of 8 months (Supplementary Figure S2).

Aβ expression in the retinas of 3xTg-AD mice was detected by two amyloid antibodies, anti-Aβ1-42 and anti-Aβ17-24 (4G8) at the ages of 6 and 8 months, when significant functional changes are found in AD retina. No extracelluar Aβ was detected at either time point. Accumulation of intraneuronal Aβ was found in the ganglion cell layer (GCL) and inner nuclear layer (INL) of the retinas of 3xTg-AD mice at 8 months of age (Figures 1D,E). Increased Aβ expression was predominately located in the soma of neurons. Aβ staining was not observed in the retinas of age-matched WT control mice.

### LBE Feeding Preserved Retinal Function of 3xTg-AD Mice

**Figure 2:**
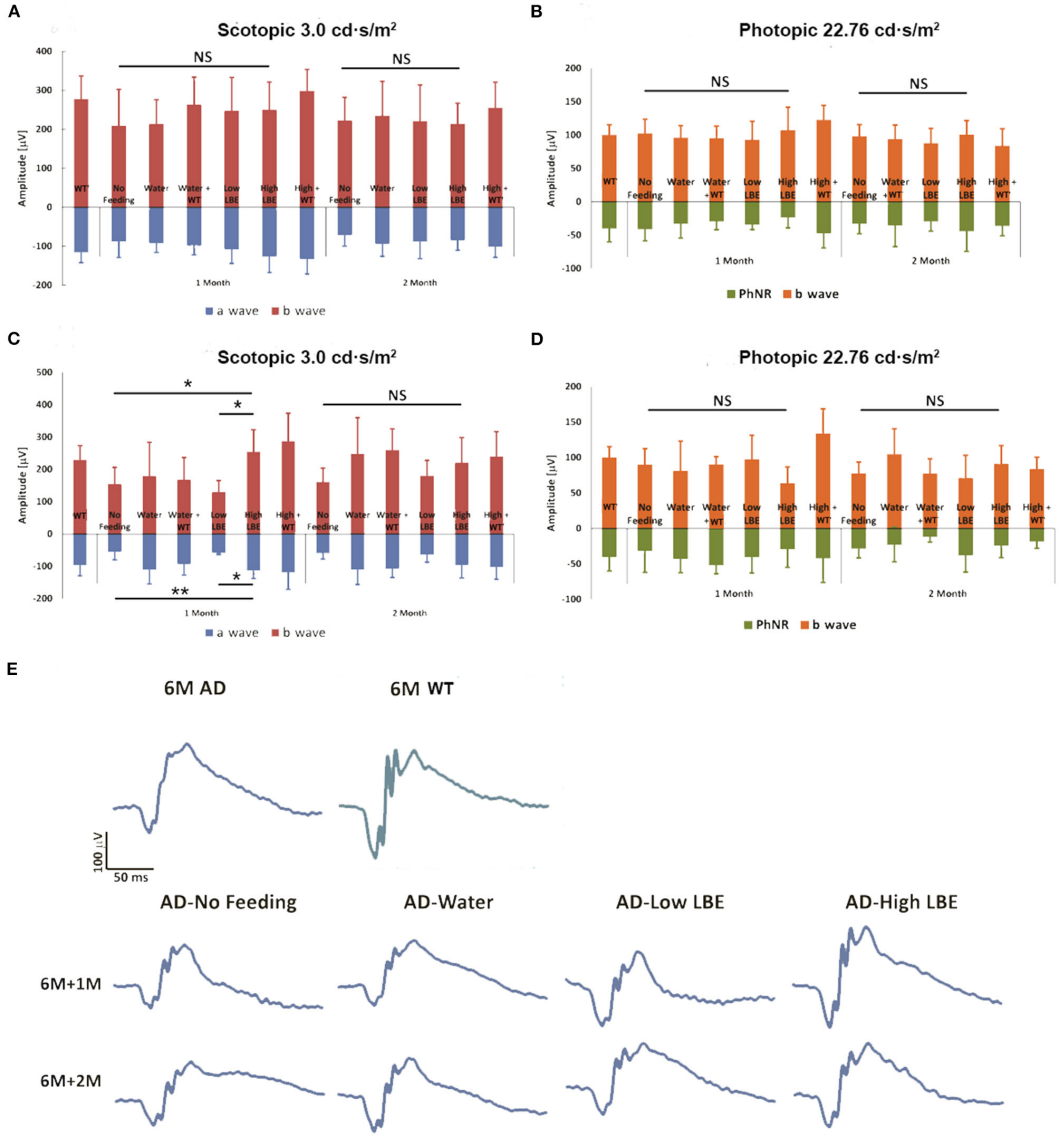
Effect on retinal function of LBE oral feeding started at different ages. LBE feeding started at 4 months or 6 months of age in both non-Tg and 3xTg-AD mice. For two months, two doses [low (200 mg/kg) or high (2 g/kg)] of LBE were given daily, while water served as the control and non-feeding as the age-matched AD control. LBE feeding from age 4 months showed a trend toward better scotopic retinal response for both a-wave and b-wave in 3xTg-AD mice **(A)**. When started at 6 months of age, high dose LBE feeding of 3xTg-AD mice for 1 month showed significantly higher retinal response to scotopic light intensity (3.0 cd·s/m^2^) compared to 3xTg-AD mice given only water **(C)**. Photopic b-wave and PhNR, indicating the function of the inner nuclear layer and RGCs, showed no differences among all the groups **(B,D)**. *p < 0.05, **p < 0.01; NS, no significant change. Typical ERG wave forms of various groups at 1 month and 2 months after the start of feeding **(E)**.

The 3xTg-AD mice begin to show impairment on the Morris water maze and inhibitory avoidance, coincident with the onset of intracellular Aβ in the brain at age 4 months. Extracellular Aβ is detectable in the cortex at 6 months of age (Billings et al., 2005). The ages at which feeding was started in this study were chosen as the ages when intracellular (4th month) and extracellular (6th month) Aβ become detectable in the brain. Our previous study using the early onset 5xFAD mouse model found that the retinal function in dark conditions (scotopic response) was enhanced by LBE at 20 g/kg oral feeding (Zhang et al., 2021). In the current study of 3xTg-AD mice, lower doses were chosen because less Aβ accumulation was reported in these mice than in the 5xFAD model. The ERG test was performed at 1 or 2 months after feeding started. As shown in Figures 2A,B, no significant changes in scotopic or photopic retinal function were induced by LBE feeding when the feeding started at age 4 months. When the feeding started at age 6 months, 1 month of 2 g/kg LBE gavage significantly increased both a-wave and b-wave amplitude in scotopic ERG compared to the no-feeding 3xTg-AD mice. The same tendency of a-wave and b-wave preservation was observed after 2 months of 2 g/kg LBE feeding, but without statistical significance (Figures 2C,E). No significant changes in photopic b-wave or PhNR were observed in LBE-fed 3xTg-AD mice (Figure 2D). At 8 months of age, morphometric analysis on the neuronal cell number and Müller cell activation also remained unchanged in 3xTg-AD mice with LBE feeding (Supplementary Figures S1, S2).

### LBE Feeding Decreased RNA Oxidation and Activation of Calpain-2, Calpain-5 in the Retinas of 3xTg-AD Mice

**Figure 3:**
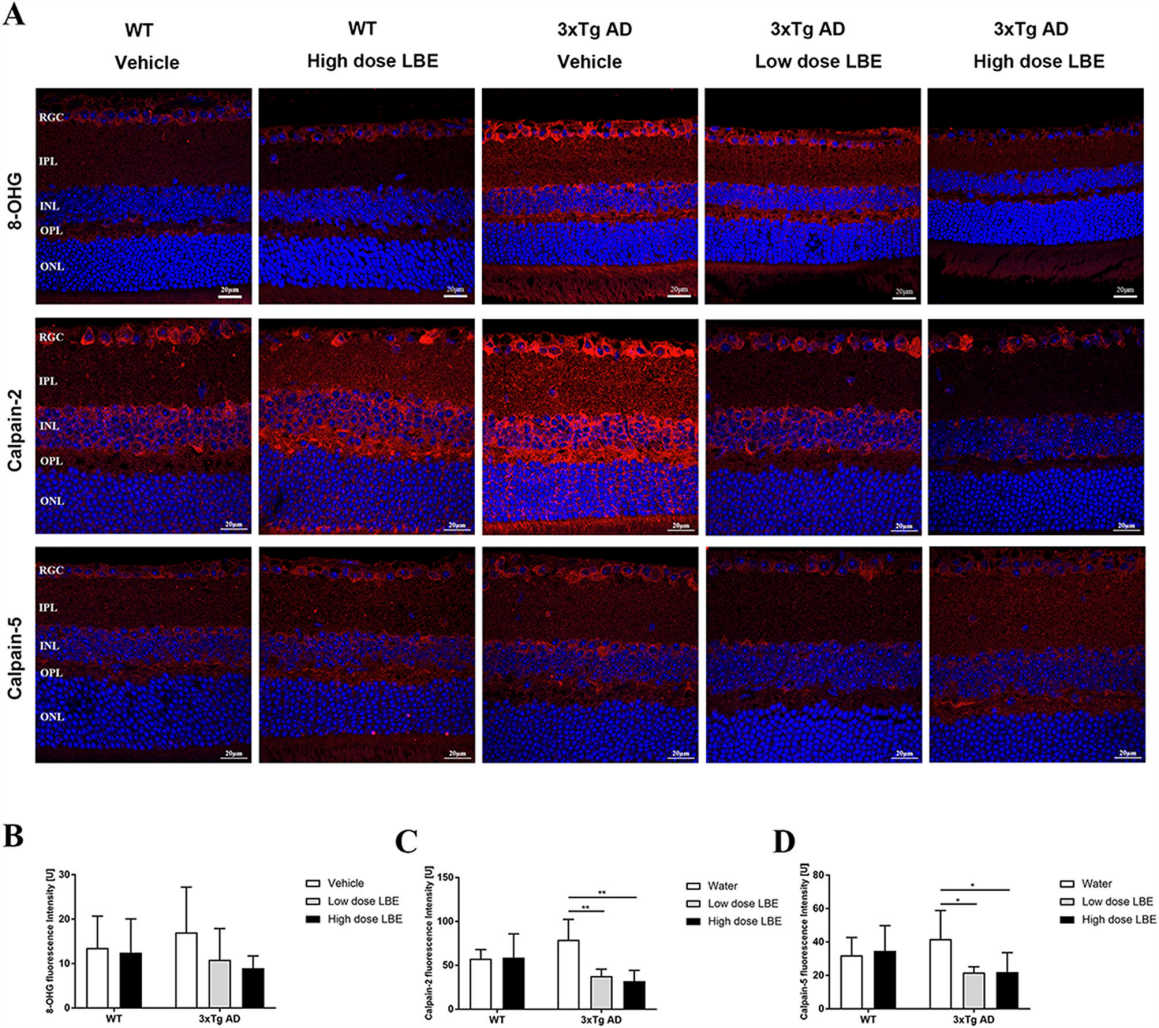
Two months LBE oral feeding downregulated expression of 8-OHG, calpain-2, and calpain-5 in 3xTg-AD mice at the age of 8 months. **(A)** Representative images of 8-OHG, calpain-2, and calpain-5 stained retinal sections of 3xTg-AD mice and age matched non-Tg control mice fed with water, low dose LBE or high dose LBE. Semi-quantification analysis showed increased 8-OHG **(B)**, calpain-2 [**(C)**, * *P* < 0.05; ** *P* < 0.01], and calpain-5 [**(D)**, * *P* < 0.05; **P < 0.01] in the retinas of 3xTg-AD mice, which could be suppressed by LBE feeding.

The guanosine oxidation product 8-hydroxyguanosine (8-OHG) is one of the most abundant and best characterized biomarkers for RNA oxidative lesions (Feyzi et al., 2007). The amount of cellular 8-OHG is a sensitive measurement of oxidative stress and is an RNA damage biomarker (Xu et al., 2021). At 8 months of age, there was an obvious trend of increased 8-OHG in the retinas of water-fed 3xTg-AD mice compared to the age matched WT controls (Figure 3A, first lane). Comparing with water-fed mice, LBE oral feeding did not change the 8-OHG level in WT mice, while reducing its level in the 3xTg-AD mice, especially with the high dose (2 g/kg) (Figure 3B).

With an increase in age, increased oxidative stress and Aβ deposition, the normally tightly controlled calpain activation regulatory system becomes impaired. Increased calpain activation is involved in the pathogenesis of AD (Mahaman et al., 2019). In the retinas of 3xTg-AD mice, the expression of both calpain-2 (Figure 3A, second lane) and calpain-5 (Figure 3A, third lane) showed a tendency toward increase compared to WT mice at 8 months of age. While there is no effect of LBE feeding comparing with water feeding in the WT mice, LBE feeding at both doses significantly decreased the expression of calpain-2 and calpain-5 in the retinas of 3xTg-AD mice (Figures 3C,D).

### LBE Feeding Preserved Impaired Pre-synaptic Densities in Retinas of 3xTg-AD Mice

**Figure 4:**
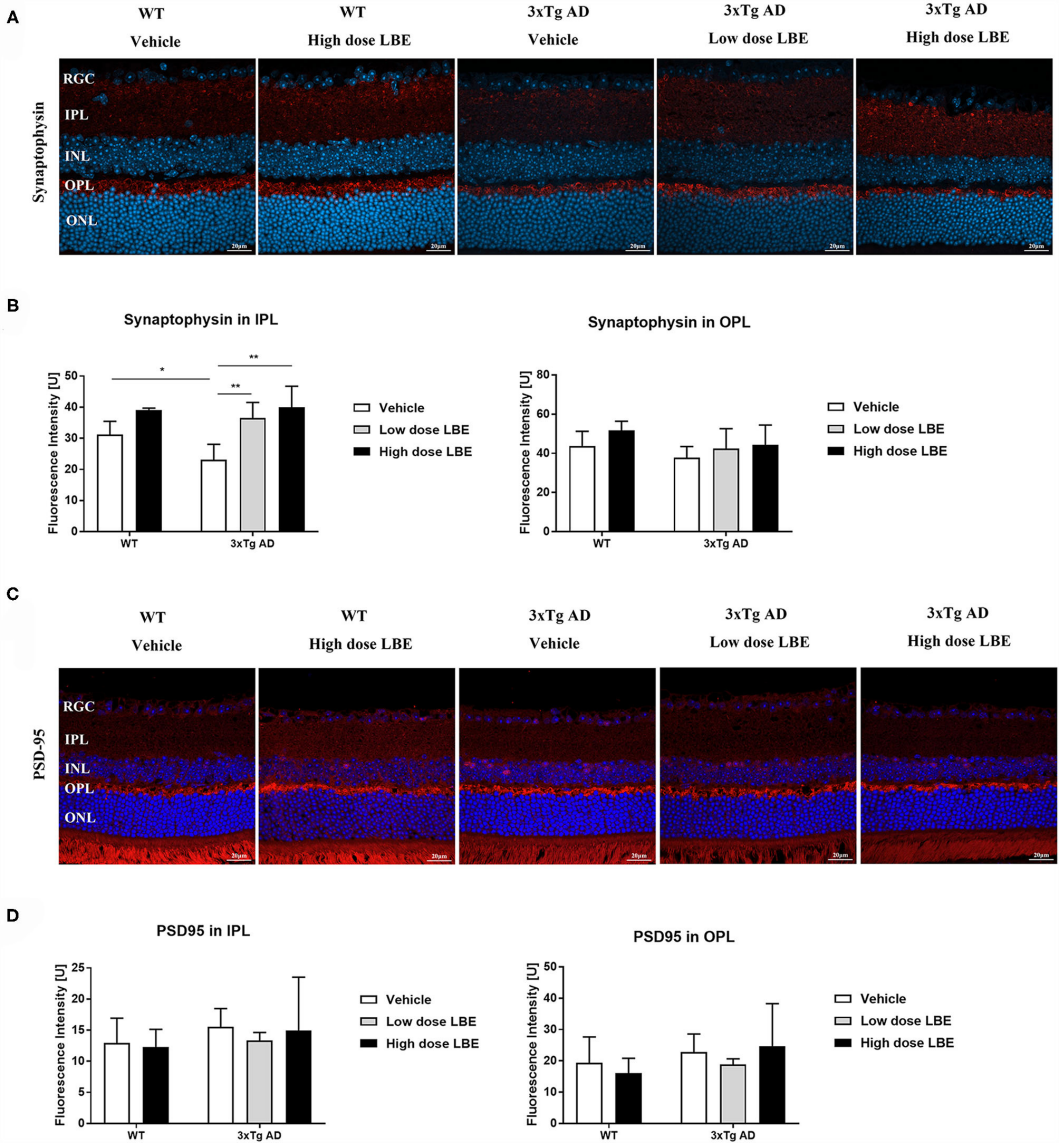
LBE oral feeding significantly preserved presynaptic terminals in 3xTg-AD mice at 8 months of age. **(A)** Representative images of retinal sections stained for the presynaptic marker, synaptophysin, in 8 month old 3xTg-AD mice and age matched non-Tg control mice fed with water, low dose LBE or high dose LBE. Semi-quantification analysis revealed decreased presynaptic densities in the IPL but not the OPL in 8 month old 3xTg-AD mice, and rescue of the decrease by high dose LBE feeding [**(B)**, * *P* < 0.05, **p < 0.01]. Representative images of retinal sections stained for the postsynaptic marker, PSD-95, in 8 month old 3xTg-AD mice and age matched wild type mice fed with water, low dose LBE or high dose LBE **(C)**. No significant changes were observed in postsynaptic densities among all the groups **(D)**.

Synaptic loss is a common pathological change in AD patients and Tg-AD models. Both pre- and post-synaptic proteins were detected in the retinas. While there were no obvious changes in the immunoreactivity of a post-synaptic protein marker (PSD-95) (Figure 4C), a pre-synaptic protein marker (synaptophysin) was significantly decreased in the retinal inner plexiform layer (IPL) of 8 month old 3xTg-AD mice (Figure 4A). The reduced synaptophysin expression level was restored by LBE feeding, with this restoration reaching significant levels at high dose (Figure 4B). Pre-synaptic protein expression in the OPL layer showed a similar but non-significant tendency. Furthermore, we explored post-synaptic changes with PSD-95 staining, and no significant changes were observed in 3xTg-AD mice compared with wild type mice (Figures 4C,D).

## Discussion

Retinal Aβ aggregation has been reported in various AD transgenic mouse models (reviewed by Chiu et al., 2012). In the current study, we used the retina as a window to study early changes in female 3xTg-AD mice. At the 8th month of age, the retinal function detected by flash ERG revealed a significant decrease of scotopic b-wave in the 3xTg-AD mice compared to WT mice. Morphometric analysis showed no significant loss of neurons in the 3xTg-AD retina. Intracellular Aβ, oxidative RNA marker 8-OHG, and calpain-2 and -5 were increased in the retinal neurons in the RGCL and the INL. The pre-synaptic protein, synaptophysin, was significantly decreased in the IPL of 3xTg-AD mice retinas. Oral feeding of LBE (2 g/kg) starting at 6 months of age decreased the activation of calpain-2 and -5 and restored the synaptophysin expression after 2 months of feeding.

As an extension of the central nervous system, the retina is considered a valuable tool for the study of CNS disorders (London et al., 2013). Accumulating evidence has revealed retinal abnormalities in AD patients, including retinal function impairment and structural changes, such as optic nerve damage, loss of ganglion cells, and retinal thinning (Chiquita et al., 2019b). These facts provided support for the idea to use the retina as a window into the AD nervous system, to help overcome the difficulty in accessing the brain and the high cost of current modalities, including PET and CSF biomarkers (Liao et al., 2018; Ngolab et al., 2019). More importantly, retinal Aβ plaques tended to show up in the retina prior to brain plaques (Liao et al., 2018). In 3xTg-AD mice, scattered extracellular Aβ deposition in the caudal hippocampus existed at the age of 6 months (Belfiore et al., 2019). In this study, intracellular Aβ immunoreactivity (4G8 or Aβ1-42) was detected in the retina at 8 months of age, together with RNA oxidation, calpain activation and reduced synaptophysin expression in the inner retina. These changes might be the reason that significant scotopic retinal function reduction in both a and b wave in the 3xTg-AD mice was detected. Morphometric analysis of the thickness change in the inner and outer retina in H&E retinal sections can be translated to the clinical detection of retinal structure using optical coherence tomography (OCT). Retinal function detected by ERG might be used as an early marker for AD diagnosis. There was no significant change in the retinal thickness using classical histopathology analysis. Moreover, no significant neuronal loss in the retina could be confirmed using immunohistochemical detection of specific cell markers such as Brn-3a and PKC. Loss of synapses in the affected brain regions correlates best with cognitive impairment in AD patients that has been considered as an early mechanism that precedes neuronal loss (Chen et al., 2019). The decrease of retinal function can be explained by the reduction of synaptic protein, especially in the inner retina in the early stage of AD.

A cytoplasmic oxidative RNA nucleoside, 8-OHG, is markedly increased within the hippocampus and temporal neocortex in AD patients at an early stage of the disease (Nunomura et al., 2009; Nunomura and Perry, 2020). Synaptic dysfunction is closely associated with oxidative stress in AD (Kamat et al., 2016). LBE has been proved to be protective against oxidative damage in various diseases (Gao et al., 2017), and to modulate glial cell function *in vitro* (Zheng et al., 2021). Two months of 2 g/kg LBE oral feeding starting at 6 months proved the effectiveness of antioxidation and preservation of synaptic function in the retina of 3xTg-AD mice. Although the preserved retinal function can only be significantly detected at the 1 month but not the two-month time point in the LBE-fed 3xTg-AD mice, an increase in animal numbers might reveal a significant effect at 2 months.

In neurodegenerative diseases including AD, abnormal activation of calpains favors Aβ accumulation and tau hyperphosphorylation in neurons and is associated with synaptic dysfunction (Trinchese et al., 2008; Medeiros et al., 2012; Diepenbroek et al., 2014). Upregulated calpain-2 and -5 were detected in the retinas of 8 month-old 3xTg-AD mice. They were down regulated by 2 months of LBE feeding, which also stabilized pre-synaptic protein expression and improved retinal function.

Gliosis is an activation of glial cells in response to central nervous system injury. In the brain tissue of 3xTg-AD mice, increased densities of active astrocytes and microglia were observed at 7 months (Caruso et al., 2013). Activation of astrocytes and Müller cells was also identified in the retinas of 3xTg-AD mice at 8 months of age (Edwards et al., 2014). However, in our study, immunochemical staining of GFAP and Iba-1 did not detect significant changes in astrocytes and microglial cell numbers, respectively, in either the brain or the retina of 8 month old 3xTg-AD mice. These findings may be explained by the fact that neuronal cell damage and extracellular Aβ deposition may be the primary inducers of glial activation in AD, but neither were present in the retinas of the 8 months old 3xTg-AD mice in the current study. Still, a possibility exists that activation of glial cells might have occurred in our mice but may have been too subtle to detect. Oligomeric Aβ induces M1 activation (pro-inflammatory) of the IMG cell line, but pretreatment with LBE shifts the cells to M2 activation (anti-inflammatory) (Sun et al., 2022). Therefore, besides the direct LBE effect on retinal neurons, modulation of retinal glial function cannot be ruled out.

A limitation of this study is that we did not identify which molecular component was responsible for the neuroprotectant activity we examined. However, we used a very simple extraction procedure to make it easy for the general population to use the same method. Therefore, it is possible that, even without pinpointing one chemical for further investigation, this LBE can be quickly and affordably adopted by more patients or even the entire aging population with the ultimate goal of reducing illness and economic burden.

## Conclusion

Our study demonstrated a protective effect of LBE feeding on synaptic function in the retinas of 3xTg-AD mice. This may be due to the ability of LBE feeding to reduce intracellular RNA oxidation and calpain-2 and -5 hyperactivation, and thus to stabilize synapses. LBE acted through multiple pathways to stabilize retinal function in 3xTg-AD mice. Daily LBE supplementation might be beneficial for neuronal function and survival even during aging in the presence of excess Aβ.

## Data Availability Statement

The original contributions presented in the study are included in the article/Supplementary Material. Further inquiries can be directed to the corresponding authors.

## Ethics Statement

The animal study was reviewed and approved by Committee on the Use of Live Animals in Teaching and Research of the University of Hong Kong.

## Author Contributions

KC, KS, and JL: conceptualization. JL, LB, SY, YL, and GX: methodology. JL and GX: data analysis. KC and KS: project administration. JL and LB: writing original draft. KC, LB, and RC: manuscript review and editing. All authors read and approved the final manuscript.

## Funding

This project was supported by Health and Medical Research Fund (HMRF, Project No: 14151281) and Midstream Research Program for Universities (MRP, Project No: MRP-092-17X) in Hong Kong to KC.

## Conflict of Interest

The authors declare that the research was conducted in the absence of any commercial or financial relationships that could be construed as a potential conflict of interest.

## Publisher's Note

All claims expressed in this article are solely those of the authors and do not necessarily represent those of their affiliated organizations, or those of the publisher, the editors and the reviewers. Any product that may be evaluated in this article, or claim that may be made by its manufacturer, is not guaranteed or endorsed by the publisher.

## Supplementary Material

Figure S1 LBE oral feeding effect on the retinal morphology of 3xTg-AD mice. **(A)** Representative images of H&E-stained retinal sections of 8 month old 3xTg-AD mice and age matched control mice fed with water, low dose LBE or high dose LBE. **(B)** Morphometric analysis revealed no significant changes in the thickness of the IRL or ONL, or in RGC numbers, in LBE-treated mice compared with water-treated 3xTg-AD mice.

Figure S2 No changes in RGC numbers, retinal bipolar cell numbers, and astrocyte/Müller cell activation were observed in 3xTg-AD mice at 8 months old. **(A)** Representative images of Brn3a, PKC-α, and GFAP stained retinal sections of 8 month old 3xTg-AD mice and age matched wild type mice fed with water, low dose LBE or high dose LBE. **(B)** RGC numbers, retinal bipolar cell numbers, and astrocyte/Müller cell activation showed no significant differences between 3xTg-AD and wild type mice, and between the LBE-treated and water-treated 3xTg-AD mice.
