# Supplementary figures and images for: Preservation of Retinal Function Through Synaptic Stabilization in Alzheimer's Disease Model Mouse Retina by Lycium Barbarum Extracts
Source: Front Aging Neurosci. 2022 Jan 13;13:788798. doi: 10.3389/fnagi.2021.788798 (PMC8792986; doi:10.3389/fnagi.2021.788798)

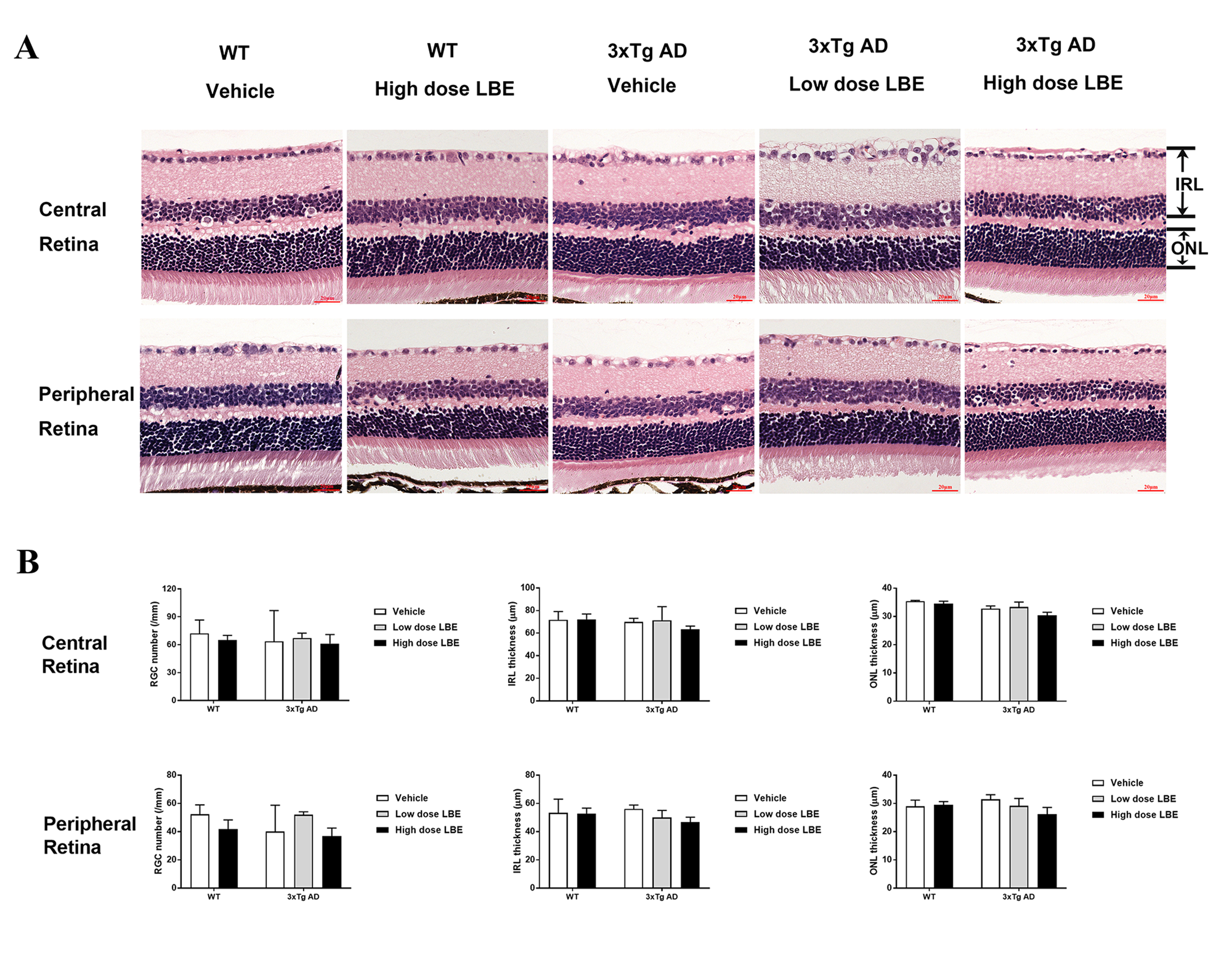

Supplement: Figure S1 — LBE oral feeding effect on the retinal morphology of 3xTg-AD mice. (A) Representative images of H&E-stained retinal sections of 8 month old 3xTg-AD mice and age matched control mice fed with water, low dose LBE or high dose LBE. (B) Morphometric analysis revealed no significant changes in the thickness of the IRL or ONL, or in RGC numbers, in LBE-treated mice compared with water-treated 3xTg-AD mice. [file Image_1.TIF]

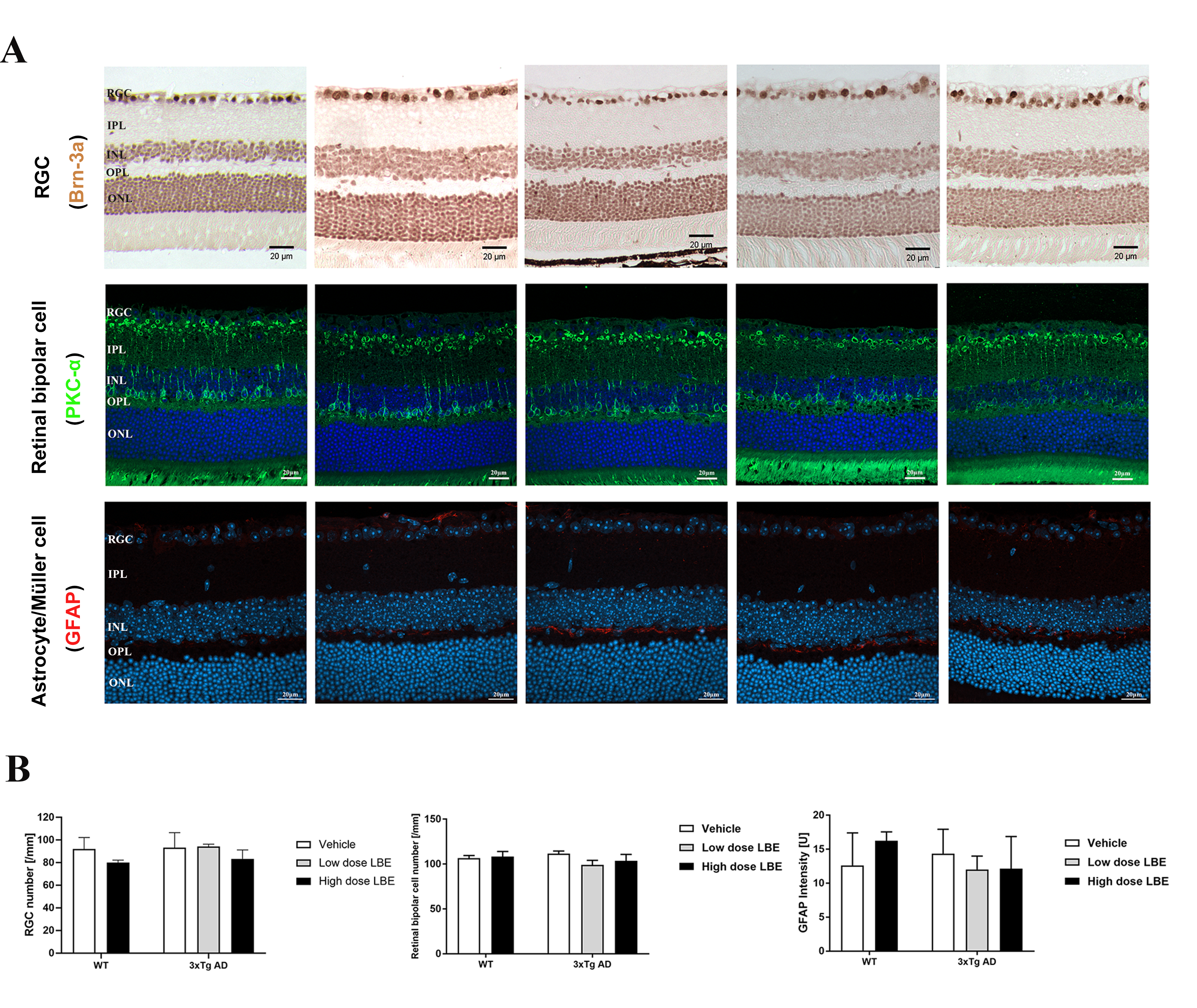

Supplement: Figure S2 — No changes in RGC numbers, retinal bipolar cell numbers, and astrocyte/Müller cell activation were observed in 3xTg-AD mice at 8 months old. (A) Representative images of Brn3a, PKC-α, and GFAP stained retinal sections of 8 month old 3xTg-AD mice and age matched wild type mice fed with water, low dose LBE or high dose LBE. (B) RGC numbers, retinal bipolar cell numbers, and astrocyte/Müller cell activation showed no significant differences between 3xTg-AD and wild type mice, and between the LBE-treated and water-treated 3xTg-AD mice. [file Image_2.TIF]
